# Combinatorial bioactive botanicals re-sensitize tamoxifen treatment in ER-negative breast cancer via epigenetic reactivation of ERα expression

**DOI:** 10.1038/s41598-017-09764-3

**Published:** 2017-08-24

**Authors:** Yuanyuan Li, Syed M. Meeran, Trygve O. Tollefsbol

**Affiliations:** 1 Department of Biology, University of Alabama at Birmingham, Birmingham, Alabama 35294 USA; 2 Comprehensive Cancer Center, University of Alabama at Birmingham, Birmingham, Alabama 35294 USA; 3 Nutrition Obesity Research Center, University of Alabama at Birmingham, Birmingham, Alabama 35294 USA; 4 Department of Biochemistry, CSIR-Central Food Technological Research Institute, Mysore, 570019 India; 5 Comprehensive Center for Healthy Aging, University of Alabama at Birmingham, Birmingham, Alabama 35294 USA

## Abstract

Conventional cancer prevention has primarily focused on single chemopreventive compounds that may not be sufficiently efficacious. We sought to investigate potential combinatorial effects of epigenetic bioactive botanicals including epigallocatechin-3-gallate (EGCG) in green tea polyphenols (GTPs) and sulforaphane (SFN) in broccoli sprouts (BSp) on neutralizing epigenetic aberrations in estrogen receptor-α (ERα) leading to enhanced anti-hormone therapeutic efficacy in ERα-negative breast cancer. Our results showed that this combinatorial treatment re-sensitized ERα-dependent cellular inhibitory responses to an estrogen antagonist, tamoxifen (TAM), via at least in part, epigenetic reactivation of *ERα* expression in ERα-negative breast cancer cells. Further *in vivo* studies revealed the combinatorial diets of GTPs and BSp significantly inhibited breast tumor growth in ERα-negative mouse xenografts, especially when combined with TAM treatment. This novel treatment regimen can lead to remodeling of the chromatin structure by histone modifications and recruitment changes of transcriptional factor complex in the *ERα* promoter thereby contributing to *ERα* reactivation and re-sensitized chemotherapeutic efficacy of anti-hormone therapy. Our studies indicate that combinatorial bioactive botanicals from GTPs and BSp are highly effective in inhibiting ERα-negative breast cancer due at least in part to epigenetic reactivation of *ERα*, which in turn increases TAM-dependent anti-estrogen chemosensitivity *in vitro* and *in vivo*.

## Introduction

The progressive loss of balance between environmental impacts and endogenous regulations in genetic/epigenetic blueprints is the primary contributor for major human pathologies such as cancers^1, 2^. Bioactive nutritives from plants appear to be crucial to correct the relationship between environmental turbulence and systematic stability that maintains human health. Studies have shown strong chemopreventive effects of several bioactive dietary compounds including (−)-epigallocatechin-3-gallate (EGCG) in green tea polyphenols (GTPs), sulforaphane (SFN) in broccoli sprouts (BSp), genistein in soybean products and resveratrol in berries on converting genetic/epigenetic abnormalities during carcinogenesis^3–8^. Human epidemiological studies and clinical trials have also provided considerable evidence for appropriate intake of these bioactive dietary compounds in prevention and therapeutic effects of multiple human malignancies including breast cancer^9, 10^. Classic mechanisms for health benefits of bioactive phytochemicals include but are not limited to mechanisms such as anti-oxidation, anti-apoptosis, anti-inflammation properties and induction/blockade of transcription factors in key signal pathways^11^. Nevertheless, recent studies demonstrate that a small portion of bioactive diets as referred to “epigenetic diets” may act as environmental regulators that shape the activity of the epigenomic profile by dynamically influencing epigenetic events such as DNA methylation and histone modifications leading to cancer chemoprevention^12^.

Among these epigenetic dietary components, we are particularly interested in EGCG in green tea polyphenols and SFN in broccoli sprouts due to their potent anti-cancer properties and robust roles in influencing epigenetic regulation. EGCG is the most abundant catechin in green tea and accounts for >50% of the total polyphenol and effective content in green tea^13^. Studies have shown that green tea polyphenol EGCG imparts its anticancer effects through various mechanisms including the induction of cell cycle arrest and apoptosis as well as inhibition of tumor metastasis and angiogenesis^14, 15^. Another mechanism of EGCG pertains to epigenetic regulation via inhibition of an important epigenetic modulator, DNA methyltransferase (DNMT), leading to correction of excessive hypermethylation in cancer cells^16^. EGCG not only reactivates tumor suppressor genes such as *p16* ^*INK4b*^ by inhibiting hypermethylation, but also inhibits tumor promoting genes such as human telomerase reverse transcriptase (*hTERT*) that contributes to its anti-cancer properties^17, 18^. EGCG can also regulate gene expression through histone modifications^5, 19^. SFN is an isothiocyanate often found in cruciferous vegetables such as broccoli sprouts and cabbage that can reduce the risk of developing many common cancers through several mechanisms including the induction of cell cycle arrest, apoptosis and phase 2 detoxification enzymes^20–22^. Interest in SFN chemoprevention effects has recently surged due to its potent activity against a key modulator of histone modification, histone deacetylase (HDAC), which leads to an increase in the global and local histone acetylation resulting in subsequent gene transcription activation^23^. Many cancer prevention studies have primarily focused on singly-administered chemopreventive compounds and relatively few studies have focused on the combined potential of bioactive botanicals due to perceived concerns with respect to additive adverse effects and potential interaction between the compounds. One way to ascertain which agents to combine is to select compounds that have similar favorable biological effects but different mechanisms for carrying out these effects. Thus, potent anti-cancer effects of EGCG and SFN as well as their important roles in regulation of epigenetic pathways via primarily influencing DNA methylation and histone acetylation, respectively, render these bioactive compounds as excellent candidates for combinatorial chemopreventive strategies^24^.

Our previous studies have shown that singular treatment with EGCG or SFN inhibits breast cancer initiation by epigenetically mediating many tumor-related gene expressions such as *hTERT* and estrogen receptor-α (*ERα*)^5, 6, 18^. We have focused on studying *ERα* transcription regulation in the current study because the status of *ERα* is an important biomarker in breast cancer. ERα(−) breast cancer has a poor prognosis due in part to the lack of target-directed approaches^25, 26^. More than 25% of ERα(−) breast cancer cells have aberrant methylation of the *ERα* promoter and histone acetylation/deacetylation have also been implicated as common mechanisms for *ERα* gene transactivation/repression in human breast cancer cells^27, 28^. Although our previous studies indicate treatment with either EGCG or SFN alone beneficially reactivates *ERα* expression, these effects are enhanced when combined with certain epigenetic modifying drugs such as 5-azacytidine, a demethylation agent, and Trichostatin A (TSA), a histone acetylation enhancing agent^5, 29^. Since 5-azacytidine and TSA are not applicable in cancer prevention, the use of epigenetic-modifying EGCG in green tea and SFN in broccoli sprouts that have similar effects, but no toxicity to normal cells or animals when used in combination at optimal concentrations, would be crucial and practical in human breast cancer prevention.

In the present investigation, we analyzed potential epigenetic mechanisms of combinatorial treatment with EGCG from green tea and SFN from broccoli sprouts on *ERα* reactivation and how this change re-sensitized ER(−) breast cancer cells to conventional anti-hormone chemotherapeutic agents such as tamoxifen (TAM) both *in vitro* and *in vivo*. We found that combinatorial treatment with these bioactive botanicals resulted in epigenetic reactivation of *ERα* leading to increased TAM-dependent anti-estrogen chemosensitivity *in vitro* and *in vivo*. This study will facilitate more effective uses of combinatorial epigenetic dietary approaches to refractory ERα-negative breast cancer, which will provide more effective options in breast cancer prevention and therapy.

## Results

### Combinatorial treatment with EGCG and SFN synergistically inhibited cell proliferation and induced cellular response to anti-hormone treatment in ERα(−) breast cancer cells

**Figure 1:**
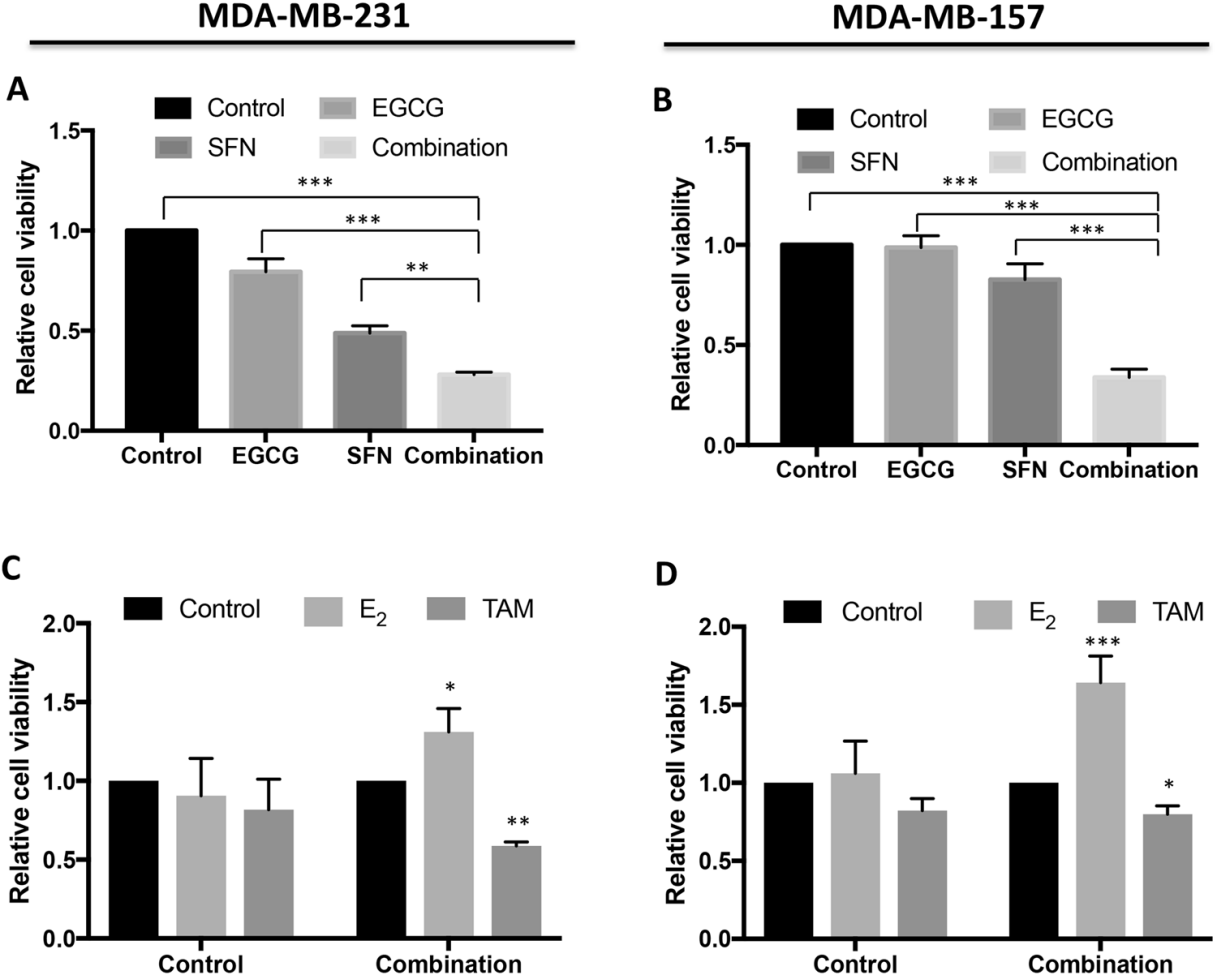
Combinatorial treatment with EGCG and SFN induced growth inhibitory effects in ERα-negative breast cancer cells. (**A** and **B**) Graphic presentation of relative cellular viability in response to treatments with EGCG and SFN, alone or in combination. ERα-negative breast cancer MDA-MB-231 (left panel) and/or MDA-MB-157 cells (right panel) were plated in 96-well plates in triplicate and treated with EGCG (20 μm) and SFN (10 μm), alone or combination for 3 days. C and D, Cellular viability in response to E_2_ and tamoxifen (TAM). EGCG and SFN-pretreated cells were treated with or without 10 nM of E_2_ or 1 µM TAM for 1 day. Control cells were grown in parallel with the treated cells but received vehicle DMSO. Data were in triplicate from three independent experiments and normalized to the control. Columns, mean; Bars, standard deviation, SD; *p < 0.05; **p < 0.01; ***p < 0.001, significantly different from the indicated comparisons.

To elucidate the combinatorial efficacy of the green tea polyphenol EGCG and broccoli sprouts SFN, we initiated our study to determine the optimal dose of combined EGCG and SFN treatment that will induce synergistic cellular growth inhibition in ERα-negative breast cancer MDA-MB-231 and MDA-MB-157 cells (Fig. S1). As shown in Fig. 1A,B, combinatorial treatment at the concentrations of 20 µM EGCG and 10 µM SFN resulted in synergistic effects on inhibiting cell proliferation in both MDA-MB-231 and MDA-MB-157 cells. This combinatorial treatment did not cause toxic effects on cell viability in normal human mammary epithelial cells (HMECs) as our studies have shown previously^24^, indicating this treatment is safe. We therefore chose optimal combinatorial treatment concentrations of 20 µM EGCG and 10 µM SFN for our subsequent studies due to a high physiological availability of these dietary compounds compared to human daily consumption, which is also consistent with our previous studies in an established cellular cancer transformation system^24^. In fact, this dietary combination regimen using relatively low concentrations of EGCG and SFN compounds shows great practical and translational potential for future human clinical trials as it represents only less than a half-cup of green tea or ~18 g BSp/serving consumed per day by an adult human^30, 31^.

Our previous studies have shown that many bioactive epigenetic diets can enhance anti-estrogen chemosensitivity in ER-negative breast cancer cells^5, 7, 8, 32^. We therefore sought to investigate whether this novel dietary combinatorial treatment with EGCG and SFN can induce hormone response in estrogen-resistant ERα-negative breast cancer cells. As expected, untreated MDA-MB-231 and MDA-MB-157 cells have no significant response to an ER pathway agonist, 17β-estradiol (E_2_), and an estrogen antagonist, tamoxifen (TAM), with respect to cellular proliferation (Fig. 1C,D). Although the combination treatment resulted in a significant reduction in cell growth, this inhibitory effect was attenuated by E_2_ treatment but strengthened in response to TAM in both ERα-negative breast cancer cell lines, especially in MDA-MB-231 cells. These results suggest that combinatorial treatment with green tea EGCG and broccoli sprouts SFN may re-sensitize ERα-negative breast cancer cells to hormonal treatment by inducing a functional ER signal pathway via at least partial reactivation of *ERα* re-expression.

### Combined treatment with EGCG and SFN induced synergistic ERα reactivation in ERα-negative breast cancer cells

**Figure 2:**
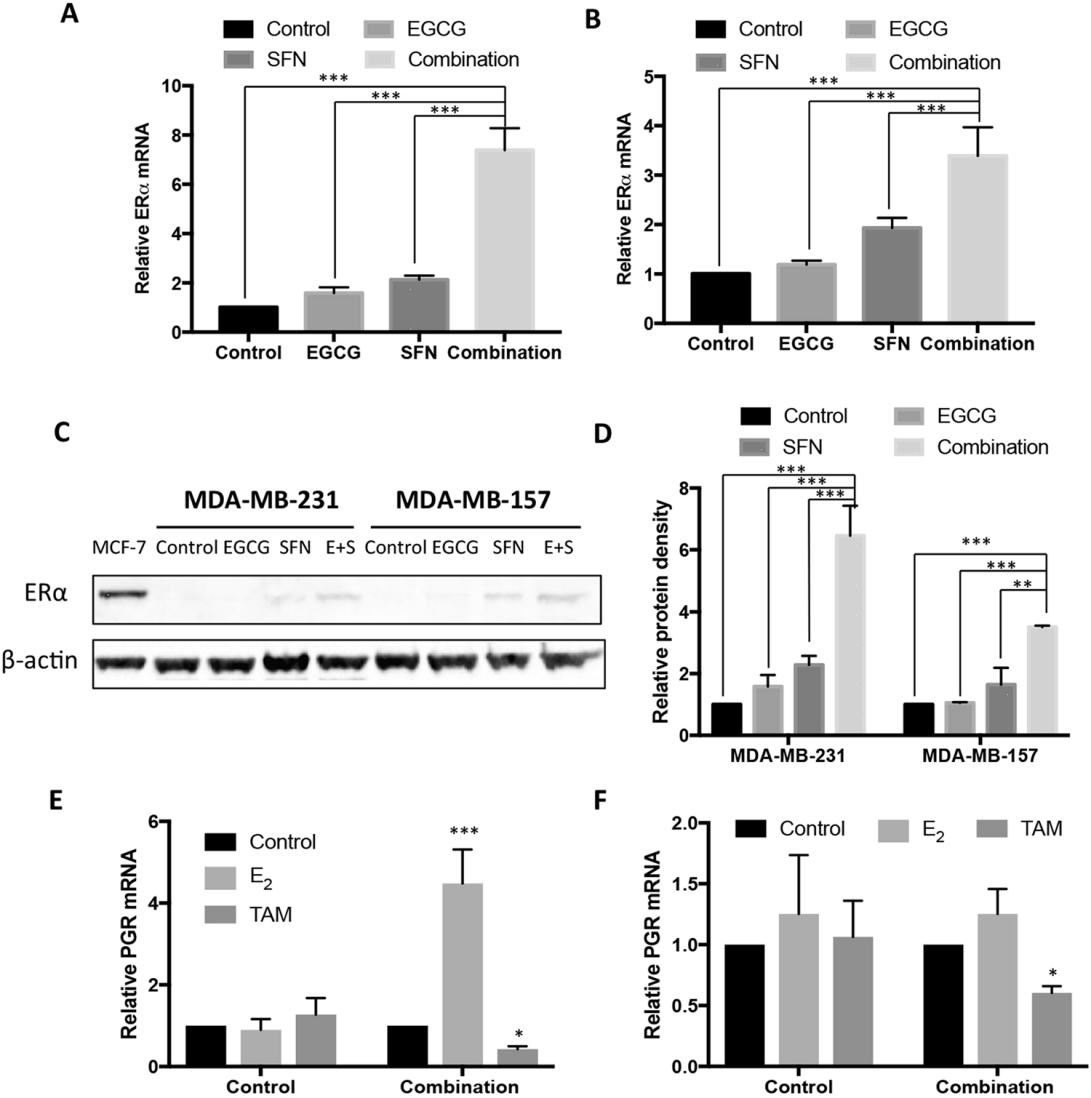
Combined treatment with EGCG and SFN induced synergistic ERα expression in ERα-negative breast cancer cells. ERα-negative breast cancer MDA-MB-231 (**A**) and MDA-MB-157 cells (**B**) were treated with EGCG and SFN, alone or combination as indicated above and relative *ERα* mRNA expression was analyzed by quantitative real-time PCR. (**C**) Protein expression of ERα in MDA-MB-231 and MDA-MB-157 cells. MCF-7 cells served as a positive control. The full-length blots were shown in the Fig. S4. (**D**) Protein quantification for (**C**). (**E**) and (**F**) mRNA expression changes of ER-responsive downstream gene, *progesterone receptor* (*PGR*), in response to E_2_ or TAM stimulation in MDA-MB-231 (**E**) and MDA-MB-157 cells (**F**). Data were in triplicate from three independent experiments and normalized to internal control and calibrated to levels in untreated samples. E + S, EGCG and SFN in combination; Columns, mean; Bars, SD; *p < 0.05; **p < 0.01; ***p < 0.001, significantly different from the indicated comparisons.

Our previous studies have shown that *ERα* could be an important molecular target for many bioactive compounds with epigenetic regulatory properties^5, 7, 8, 32^. We next sought to determine whether optimal combinatorial treatment with EGCG and SFN can induce *ERα* re-expression in ERα-negative breast cancer cells. Our results showed that EGCG or SFN treatment alone or in combination resulted in significant *ERα* transcriptional activation (*p* < 0.001) in both ERα-negative breast cancer cells such as MDA-MB-231 cells (Fig. 2A) and MDA-MB-157 (Fig. 2B), but combination treatment induced a synergistic effect of *ERα* reactivation compared to the individual treatment of EGCG or SFN, especially in MDA-MB-231 cells.

We further investigated protein expression for treatments with EGCG and SFN alone or together in ERα-negative breast cancer MDA-MB-231 and MDA-MB-157 cells. ERα-positive MCF-7 breast cancer cells served as a positive control. As shown in Fig. 2C for actual protein image and Fig. 2D for protein quantification based on band density, our results indicated that combinatorial treatment induced ERα protein synergistic re-expression in ERα-negative breast cancer cells, which was consistent with mRNA expression as shown Fig. 2A,B. These results indicate that the combination of EGCG and SFN may induce enhanced anti-estrogen chemosensitivity through *ERα* re-activation in hormone-resistant breast cancer cells. Although our combination treatment can induce *ERα* re-activation in ER-negative breast cancer cells, it is still important to demonstrate the effective response of this combination by testing gene transcriptional change of ER-responsive downstream gene, *progesterone receptor* (*PGR*)^33^, in response to E_2_ or TAM stimulation. Our results showed that the combinatorial treatment with EGCG and SFN resulted in significant transcriptional activation/repression in ER downstream *PGR* expression in response to E_2_ and TAM, respectively, in both ERα-negative MDA-MB-231 (Fig. 2E) and MDA-MB-157 cells (Fig. 2F). These results were consistent with cell viability changes shown in Fig. 1C,D, suggesting this combinatorial treatment induced a functional ER response in ER-negative breast cancer cells. In addition, this combinatorial dietary regimen exhibited greater efficacy in MDA-MB-231 cells than MDA-MB-157 cells by inducing more prominent cellular growth inhibition and *ERα* re-activation, indicating a potential cellular-dependent property for this regimen.

Due to the epigenetic impacts of EGCG on regulation of DNA methylation, and SFN on histone acetylation, respectively, we introduced a combination treatment with 5-aza, a demethylation agent, and TSA, a histone deacetylase (HDAC) inhibitor, as compared to the similar effects of EGCG and SFN. As shown in Supplementary Figures (Fig. S2A and B), 5-aza/TSA treatment resulted in significant *ERα* reactivation in mRNA and protein levels, especially in MDA-MB-157 cells. However, combinatorial treatment-induced *ERα* reactivation was more obvious in MDA-MB-231 cells as compared to 5-aza/TSA treatment. These results suggest that EGCG and SFN may exert similar epigenetic effects as shown in the demethylation agent and HDAC inhibitor, respectively, which may contribute to *ERα* reactivation in ERα(−) breast cancer. Considering the higher toxicity and side effects of 5-aza and TSA in clinical studies^34^, our novel combinatorial dietary regimen shows a great translational potential in future breast chemotherapeutic strategy.

### Gene expression and enzymatic activity changes of epigenetic modulators in response to EGCG and SFN treatments

To further interpret the epigenetic mechanisms on *ERα* regulation by EGCG and SFN treatment, we assessed gene expression and enzymatic activities of two important epigenetic-modulatory enzymes including HDAC1 involved in regulation of histone acetylation and DNA methyltransferase1 (DNMT1) involved in regulation of DNA methylation processes in breast cancer MDA-MB-231 and MDA-MB-157 cells. These two specific epigenetic enzymes including HDAC1 and DNMT1 were strategically selected. HDAC1 belongs to Class I HDACs family and is considered one of the most common and well-studied HDACs. Numerous studies have shown that HDAC1 regulates cellular proliferation not only in normal development but also in development of breast cancer^35–37^. DNMT1 is one of the most important DNMTs and plays major roles in maintaining DNA methylation patterns during normal development as well as breast tumorigenesis^38^. Our previous studies also shown that EGCG and a HDAC inhibitor, trichostatin A, can reactivate *ER* expression by affecting the recruitment of HDAC1/DNMT1 and other repressors in the *ER* promoter^5^. Due to these aforementioned reasons, we therefore chose HDAC1 and DNMT1 as our major investigation targets in this study.

**Figure 3:**
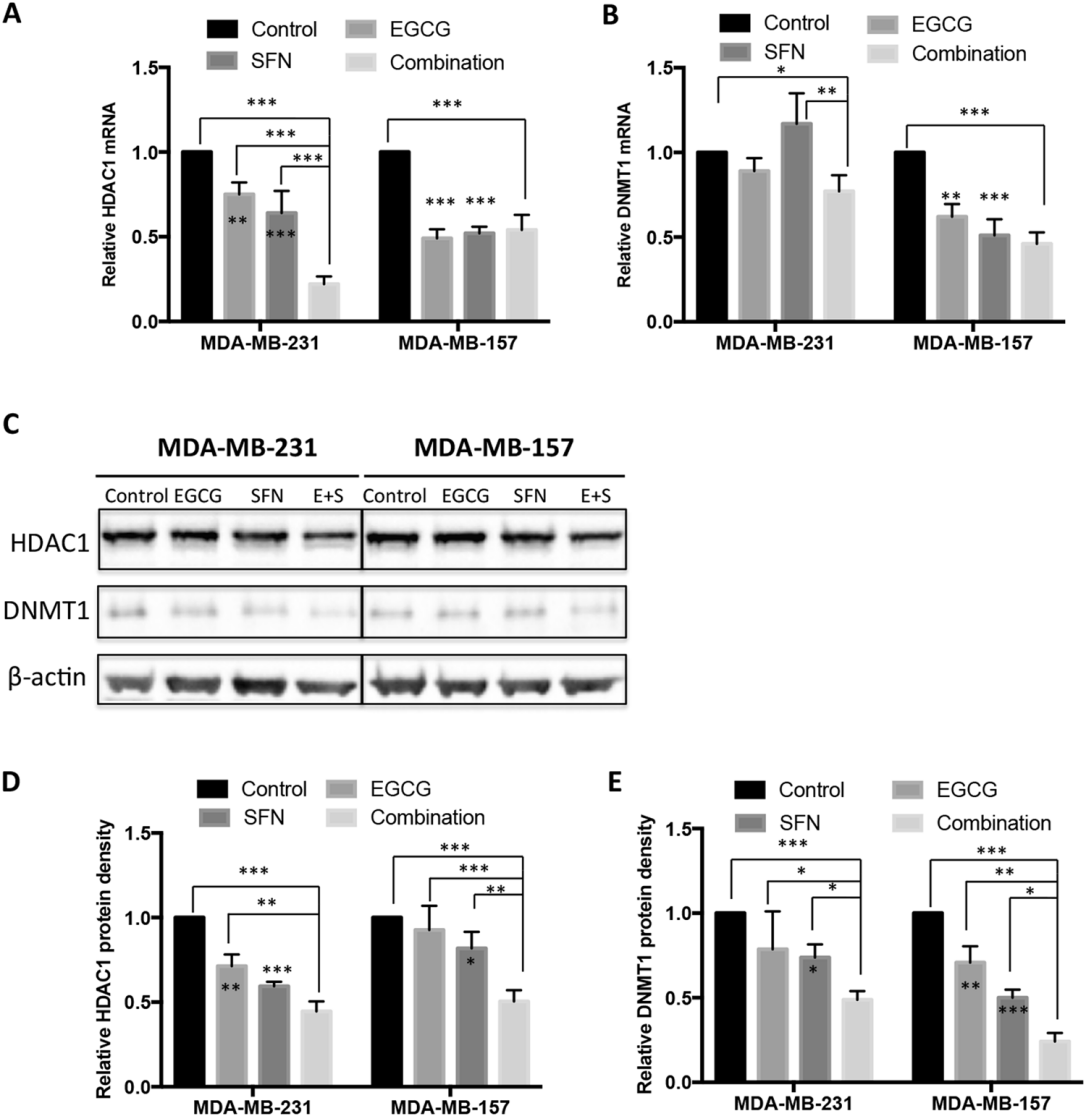
Gene expressions of HDAC1 and DNMT1 by EGCG and/or SFN treatment. Quantitative real-time PCR was performed to measure relative transcription of *HDAC1* (**A**) and *DNMT1* (**B**) in MDA-MB-231 cells and MDA-MB-157 cells. (**C**) Protein expression of HDAC1 and DNMT1 in MDA-MB-231 and MDA-MB-157 cells. β-actin was loaded as an internal control. The full-length blots were shown in the Supplementary Information. (**D**) HDAC1 protein quantification. (**E**) DNMT1 protein quantification. Data were in triplicate from three independent experiments and normalized to internal control and calibrated to levels in untreated samples. E + S, EGCG and SFN in combination; Columns, mean; Bars, SD; *p < 0.05; **p < 0.01; ***p < 0.001, significantly different from control or the indicated comparisons.

**Figure 4:**
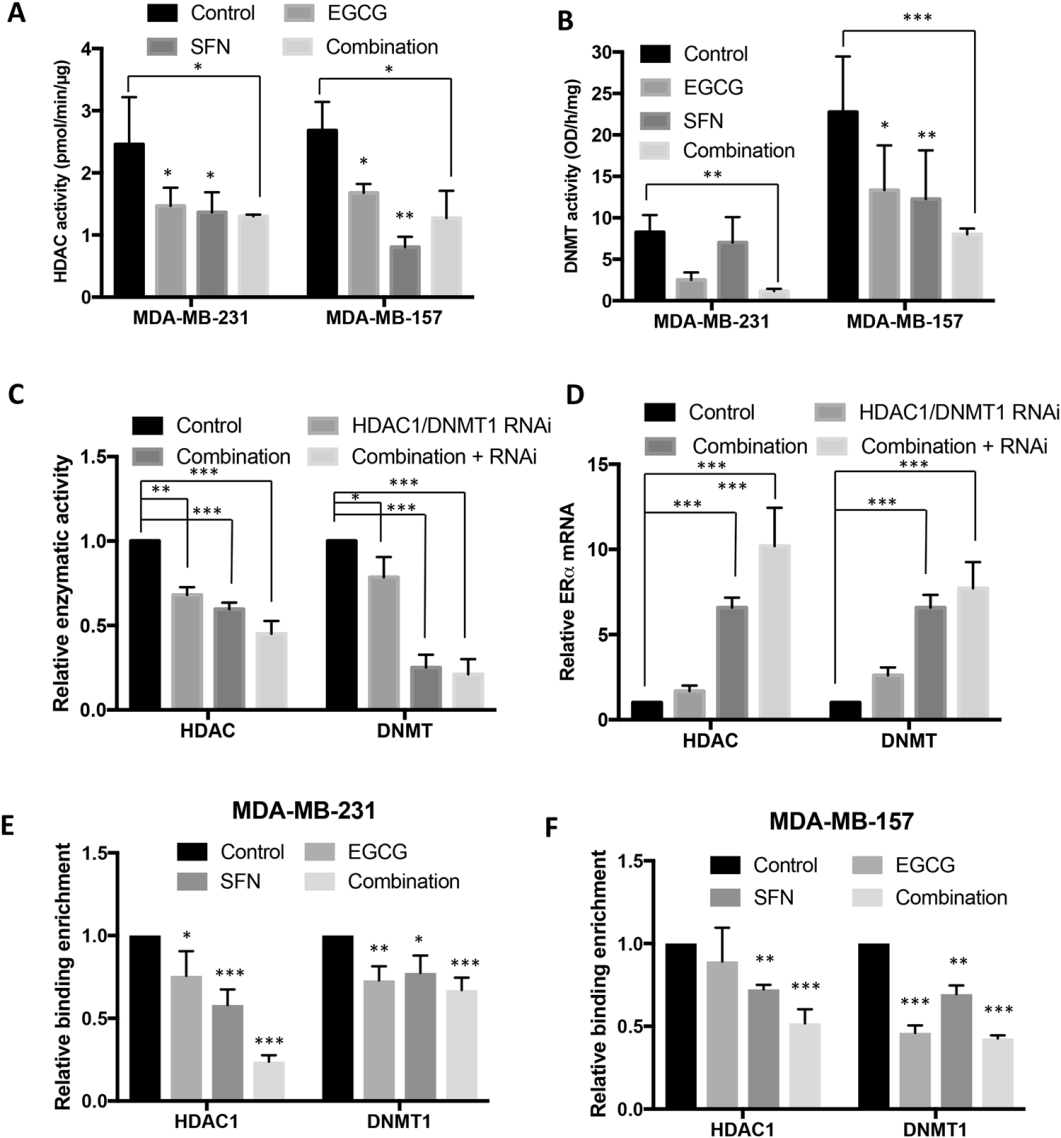
Combinatorial treatment with EGCG and SFN caused *ERα* expression changes through regulation of HDAC1 and DNMT1. (**A**) HDAC enzymatic activity. (**B**) DNMTs enzymatic activity. Nuclear proteins from MDA-MB-231 and MDA-MB-157 cells were extracted after the treatment as described above. The HDAC and DNMT activity assays were performed according to the manufacturer’s protocols. The values of enzymatic activities of HDACs and DNMTs are the means of three independent experiments. (**C**) and (**D**) EGCG and SFN-induced *ERα* transcriptional activation through affecting epigenetic pathways via directly influencing expression and enzymatic activities of HDAC and DNMT. (**C**) Relative HDAC enzymatic activity (left panel) and DNMT enzymatic activity (right panel) when expressions of HDAC1 or DNMT1 were reduced. (**D**) Relative *ERα* mRNA expression. Combination-treated or untreated MDA-MB-231 cells were transfected with either *HDAC1* or *DNMT1* siRNA to inhibit related gene expression and extracted nuclear protein or RNA after three days of transfection. (**E**) and (**F**) binding abilities of HDAC1 and DNMT1 in the *ERα* promoter were determined by ChIP assay in the MDA-MB-231 (**E**) and MDA-MB-157 cells (**F**) in response to EGCG and/or SFN treatment. Data were in triplicate from three independent experiments and normalized to internal control and calibrated to levels in untreated samples. Columns, mean; Bars, SD; **p < 0.01; ***p < 0.001, significantly different from control.

As shown in Fig. 3A, either single or combinatorial treatment with EGCG and/or SFN can significantly reduce gene expressions of *HDAC1*. In addition, the combination induced a synergistic effect in *HDAC1* mRNA reduction in MDA-MB-231 cells rather than MDA-MB-157 cells. The patterns of HDAC1 protein expression were similar as seen in its transcription levels in MDA-MB-231 cells in response to EGCG and/or SFN treatment (Fig. 3C,D). In MDA-MB-157 cells, combinatorial treatment with EGCG and SFN significantly reduced HDAC1 expression in protein level (*p* < 0.001) rather than in mRNA level, suggesting that this combination regimen more likely affects translational regulation of HDAC1 than transcriptional control in these cells. Consistently, we observed similar effects on HDAC enzymatic activity in response to combined treatment with EGCG and SFN (Fig. 4A). These results indicate that this dietary regimen may be more effective in regulation of HDAC1 expression and histone acetylation in breast cancer MDA-MB-231 cells than MDA-MB-157 cells, which shows a differential cellular response to this combination treatment.

Our results regarding *DNMT1* mRNA transcription (Fig. 3B) as well as protein translation levels (Fig. 3C,E) suggested that the combination of EGCG of SFN is necessary to induce more effective impacts on gene transcriptional activation of DNMT1 in both of the tested cell lines. Interestingly, it is likely that this combinatorial dietary regimen is more effective in regulation of DNMT1 expression in breast cancer MDA-MB-157 cells than MDA-MB-231 cells, which is opposite to what we have found in HDAC1 expression. Further studies revealed that MDA-MB-157 cells had higher endogenous DNMT activity than MDA-MB-231 cells (Fig. 4B), which may partially explain why MDA-MB-157 cells exhibited a better response targeting DNMT activity by the dietary treatments. Collectively, these results indicate that the combination of EGCG and SFN treatment may preferably affect different epigenetic pathways in different cell lines. In particular, this dietary regimen may exert its anti-breast cancer properties most likely via influencing histone acetylation in breast cancer MDA-MB-231 cells and DNA methylation in MDA-MB-157 cells.

### EGCG and SFN affected ERα expression through directly influencing epigenetic pathways via regulation of HDAC1 and DNMT1

To further verify the direct roles of epigenetic mechanisms in regulation of *ERα* reactivation, we extended our study to illustrate the potential correlation of regulation of important epigenetic modulators, HDAC1 and DNMT1, with *ERα* reactivation in response to combinatorial treatment with EGCG and SFN. For this more in depth mechanistic analysis, we focused on ERα(−) breast cancer MDA-MB-231 cells. We first tested whether the combination of EGCG and SFN affects epigenetic pathways via directly influencing expression of HDAC1 or DNMT1. We found that combinatorial treatment induced decreased enzymatic activities in HDACs (Fig. 4C, left panel) and DNMTs (Fig. 4C, right panel), which was consistent with our abovementioned findings. Furthermore, knockdown of HDAC1 or DNMT1 expressions by the relevant RNAi also showed a similar trend in down-regulation of HDACs and DNMTs activity and this effect was further strengthened when combined with EGCG and SFN treatment, which suggested that this combinatorial regimen affects epigenetic pathways via directly influencing expression of HDAC1 or DNMT1 and subsequent changes in enzymatic activities leading to changes in chromatin structures and gene expression profiles. This result was further verified in a separate experiment by evaluating HDAC or DNMT activity using EGCG and SFN post-treated control nuclear protein (Fig. S3). Similarly, combinatorial treatment with EGCG and SFN significantly reinforced *ERα* transcription increment when two epigenetic modulators, HDAC1 (Fig. 4D, left panel) and DNMT1 (Fig. 4D, right panel), were suppressed. These results further indicate that combined EGCG from green tea polyphenols and SFN from broccoli sprouts are highly effective in inhibiting breast cancer development by, at least in part, regulating key tumor-related gene expression such as *ERα* through epigenetic mechanisms. To link the roles of HDAC1 and DNMT1 to their transcriptional regulation in *ERα* reactivation, we tested binding ability changes of these two transcriptional modulators by ChIP assay and found that dietary EGCG and SFN can cause significant reduction of binding of HDAC1 and DNMT1 to the *ERα* promoter in both ER-negative breast cancer MDA-MB-231 and MDA-MB-157 cells, especially when these two compounds were combined together (Fig. 4E,F). Our results highlight important mechanisms that are potentially involved in bioactive dietary components EGCG and SFN-induced *ERα* reactivation in ER-negative breast cancer cells through affecting HDAC1 and DNMT1-mediated epigenetic pathways as well as transcriptional regulation via impairing the binding abilities of transcriptional repressors to the *ERα* promoter.

### Combinatorial treatment with orally-fed GTPs and BSp inhibited tumor growth and enhanced chemosensitivity of TAM in ERα(−) mouse xenografts

Because we found that combinatorial treatment with green tea polyphenol EGCG and broccoli sprout component SFN led to functionally ERα reactivation in ERα-negative breast cancer cells *in vitro*, we speculated that this change may benefit further therapeutic strategies through re-sensitizing hormone-resistant ERα(−) breast cancer cells to a hormone antagonist such as TAM. We therefore extended our studies to determine the *in vivo* breast cancer inhibitory properties of this dietary administration when combined with TAM treatment. We used an orthotopic xenograft mouse model by injecting ERα(−) breast cancer MDA-MB-231 cells in the mammary pad of the athymic nude mice, and examined whether dietary administration with either GTPs and BSp alone or in combination can inhibit the growth of mouse xenografts. We fed the mice 0.3% GTPs in drinking water and/or a diet supplemented with 13% BSp two weeks prior to injection of breast cancer cells and the treatment continued throughout the study, which mimic the processes of prevention and therapeutic effects by these treatments. The concentrations of the diets used in this study are converted from our *in vitro* results corresponding to drinking 1-2 cups (1.5 mg EGCG/ml water) of green tea and consuming 133 g (~2 cups) broccoli sprout/per day by an adult human, respectively^39, 40^, which are considered physiologically achievable and has high translational potential.

**Figure 5:**
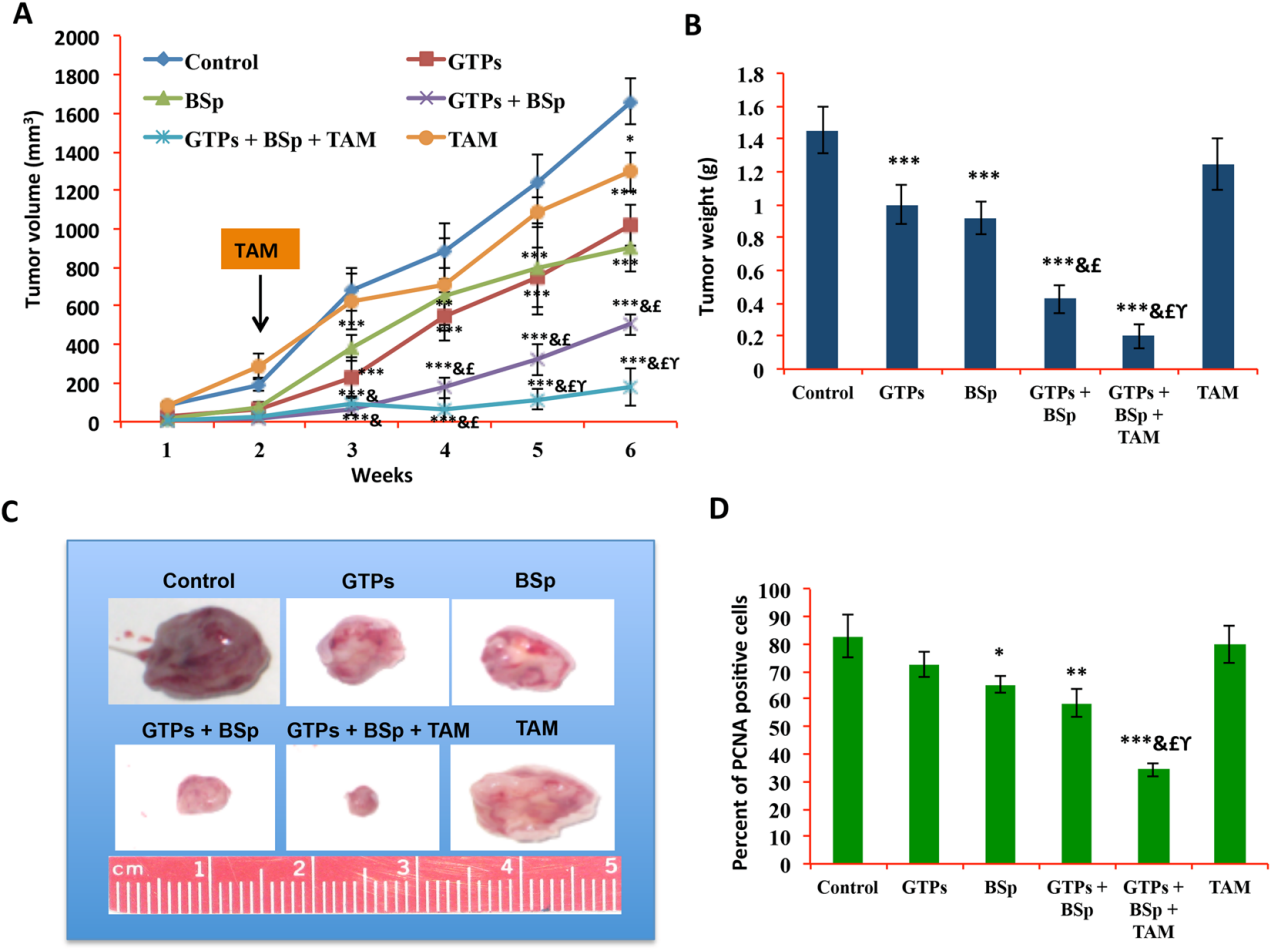
Tumor inhibitory effects of dietary GTPs and BSp with or without TAM treatment on tumor growth of ERα(−) breast cancer MDA-MB-231 xenografts. Female athymic nu/nu mice were injected with MDA-MB-231 cells. Mice (5 per group) were administered either regular control diet, 0.3% GTPs in drinking water, 13% BSp diet or two diets in combination two weeks prior to injection and thereafter. (**A**) Tumor volume. Tumor volumes were observed weekly after injection and represented as mean values for each group. One 21-day release of 25 mg TAM pellet was implanted subcutaneously two weeks post-injection as indicated with an orange box. (**B**) Tumor weight. (**C**) Representative photographs of the breast tumors in different experimental groups when harvested at the termination of the experiment (**D**) Bar chart is presented showing the immunohistochemical results in terms of percentage of PCNA-positive cells. PCNA-positive cells were counted in 5 different areas of the sections. Symbols and columns, mean; Bars, SD; *p < 0.05, **p < 0.01, ***p < 0.001, significantly different from control group; ^&^ *p < *0.01, significantly different from BSp group; ^£^ *p* < 0.01, significantly different from GTPs group; ^ϒ^ *p* < 0.05, significantly different from GTPs + BSp group.

Tumor volume studies showed that both dietary GTPs and BSp alone significantly suppressed tumor growth from as early as 3-weeks post injection (*p* < 0.001, Fig. 5A). However, combinatorial treatment with GTPs and BSp was significantly efficacious in inhibiting mouse xenograft growth compared with GTPs and BSp treatment alone after 3-weeks post injection. We also evaluated the therapeutic effect of combinatorial treatment with GTPs and BSp with the anti-estrogen chemotherapeutic agent, TAM, by administering TAM 2 weeks post-injection as indicated in Fig. 5A. As expected, TAM did not show significant effect in regression in the size of the established tumors due to its poor effect on ERα-negative breast cancer. When it was combined with GTPs and BSp treatment, TAM treatment resulted in a significant inhibition of tumor growth rate compared to the control (*p* < 0.001). More importantly, this inhibitory effect was significantly pronounced from 2 weeks after TAM administration compared to combination treatment with GTPs and BSp (*p* < 0.001) suggesting that this novel dietary combination regimen enhances the anti-tumor properties of TAM by re-sensitizing ERα-negative breast cancer to anti-hormone therapy. Consistently, studies on xenograft tumor weight showed that TAM-treated mice administered the combination diets carried the smallest tumors compared to all the other treatment groups (Fig. 5B,C).

To further analyze the potential *in vivo* anti-proliferative properties of dietary GTPs and BSp administration as well as potential therapeutic efficacy when combined with TAM, we performed immunohistochemical assays to detect PCNA-positive cells as an *in vivo* indicator for cellular proliferation in mouse xenograft tumors. As shown in Fig. 5D, PCNA-positive cells were significantly depleted by GTPs and BSp treatment alone and in combination compared to the control group. However, the percentage of PCNA-positive cells was significantly reduced in TAM-treated combination group compared to all the other treatment groups, suggesting this combinatorial regimen further consolidates the anti-tumor effect of TAM treatment by inhibiting tumor cell proliferation. Taken together, these observations reveal strong preventive and therapeutic efficacy of combinatorial treatment of GTPs and BSp against *in vivo* ERα(−) breast tumor growth and this effect is further strengthened by combination treatment with TAM.

### Dietary combination of GTPs and BSp increased ERα expression but decreased expression of HDAC1 and DNMT1 in mouse orthotopic xenografts

**Figure 6:**
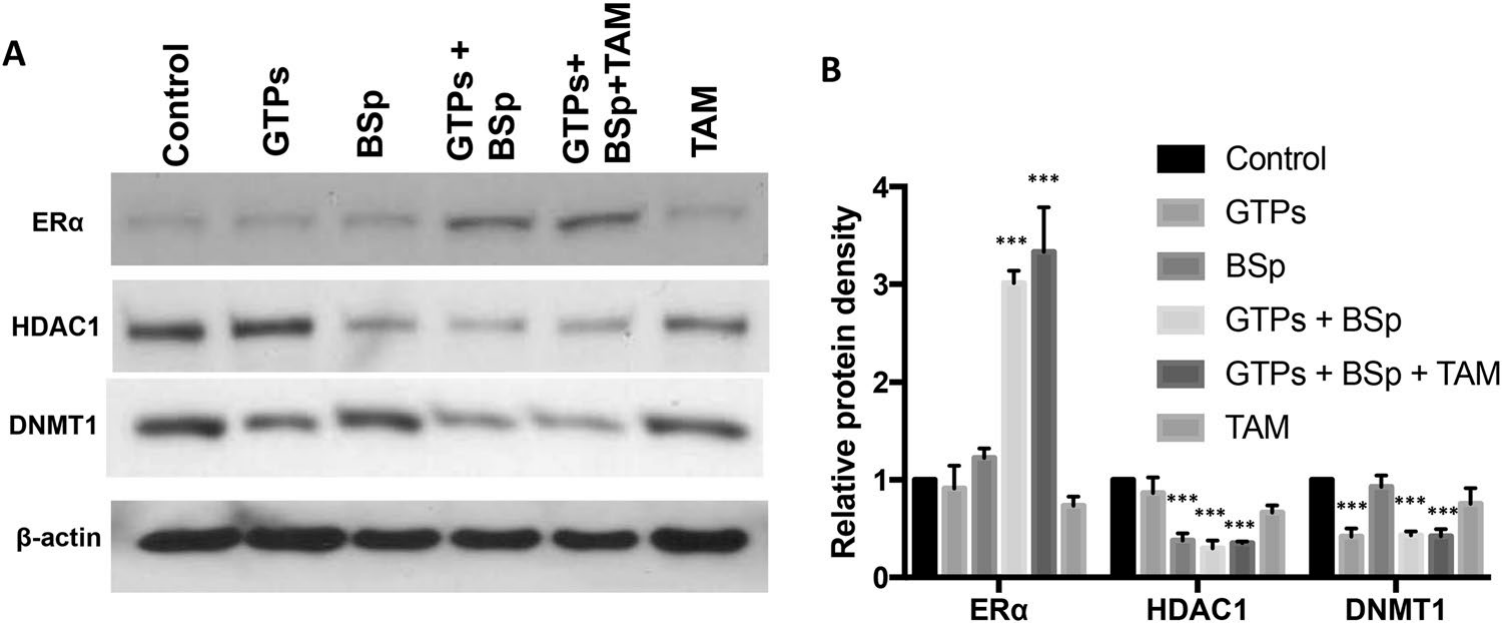
Expression changes of ERα, HDAC1 and DNMT1 in mouse orthotopic xenografts. (**A**) Protein levels of ERα, DNMT1 and HDAC1 in mouse orthotopic tumors in mammary glands using western blot analysis. The full-length blots were shown in the Supplementary Information. (**B**) Protein qualification analysis based on the band density. Data were in triplicate from three independent experiments. Relative protein expression was calculated by normalizing the original data to internal control and calibrating to levels in untreated samples. Columns, mean; Bars, SD; **p < 0.01; ***p < 0.001, significantly different from control.

In our abovementioned studies, *in vitro* dietary combination with EGCG and SFN can reactivate *ERα* expression by influencing epigenetic pathways via regulation of two important epigenetic modulators, HDAC1 and DNMT1. We therefore investigated whether combinatorial treatment with dietary GTPs and/or BSp can epigenetically impact ERα expression contributing to TAM re-sensitizing ERα-negative breast cancer *in vivo*. We evaluated ERα protein expression in mouse tumor samples and found significant ERα protein increment in the ERα(−) xenograft tumors from the combination-fed and combination + TAM-fed groups compared with the control (Fig. 6A,B), which was consistent with our previous *in vitro* observations. We further evaluated the expression status of HDAC1 and DNMT1 in mouse tumors because they are the most important epigenetic enzymes involving histone modification and DNA methylation that may influence ERα expression. As indicated in Fig. 6A,B, combination treatment with EGCG and SFN caused a significant reduction of protein expression in both epigenetic enzymes, especially when TAM was also administered. These results indicate that epigenetic mechanisms may contribute to dietary combination of GTPs and BSp-induced *ERα* re-activation leading to increased chemosensitivity of TAM therapy in hormone-resistant ERα-negative breast cancer.

### Dietary combination caused histone modification changes and altered binding ability of transcription repressor complex in the promoter region of *ERα*

**Figure 7:**
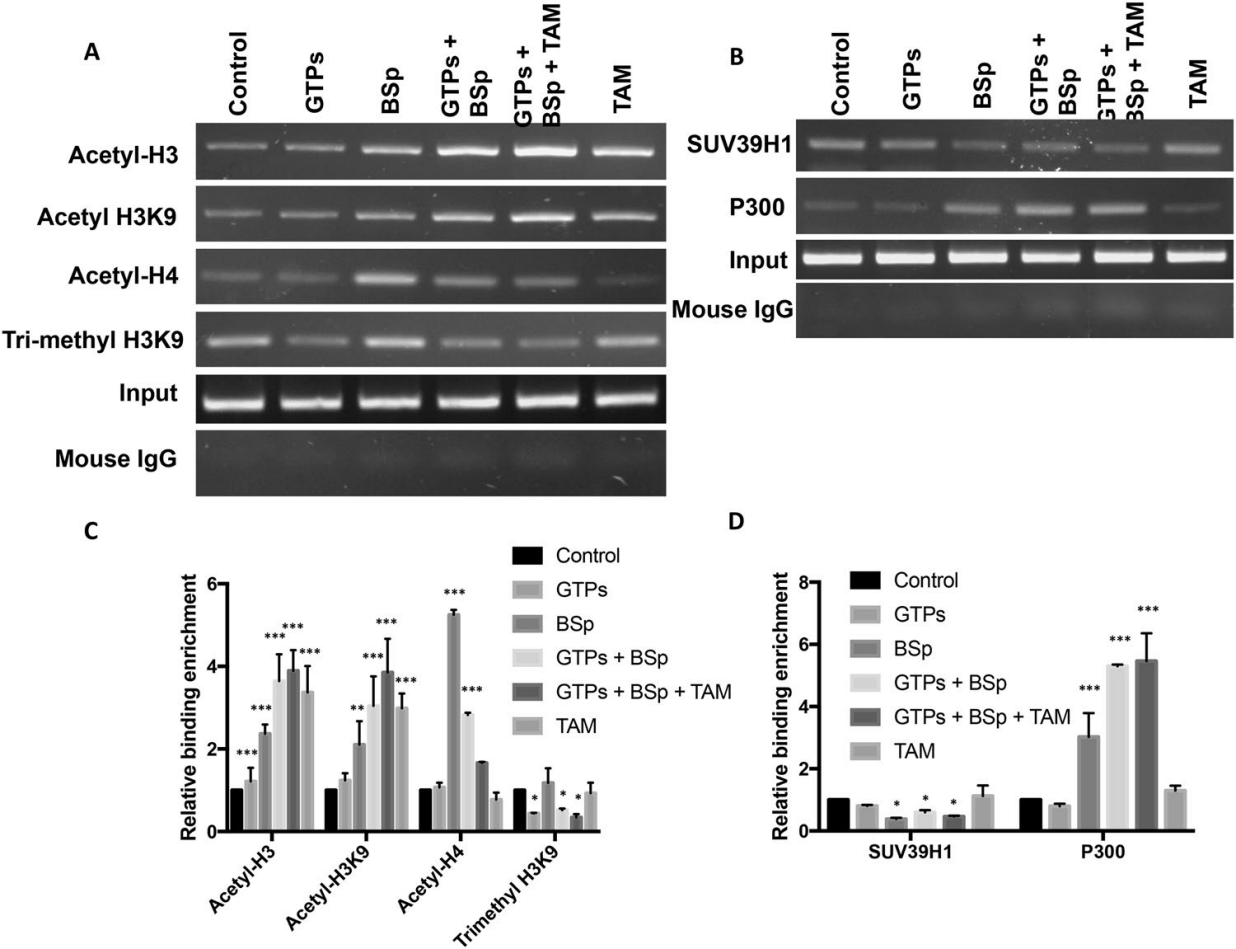
Alterations of histone modification and binding ability of transcriptional complex in the promoter region of *ERα* in the mouse orthotopic xenografts. (**A**) Histone modification patterns in the *ERα* promoter. (**B**) Binding abilities of transcriptional co-repressor, SUV39H1 and co-activator, P300, to the *ERα* promoter. Histone modification patterns and binding of transcription factors were determined by ChIP assay as described previously. Representative photograph from an experiment was repeated in triplicate. The full-length gels were shown in the Supplementary Figures (Fig. S4). Orthotopic tumors in mouse mammary glands from different treatment groups were treated as described previously and analyzed by ChIP assays using chromatin markers including acetyl-H3, acetyl-H3K9, acetyl-H4 and trimethyl-H3K9, transcription factor antibodies for SUV39H1 and P300 and mouse IgG control in the promoter region of *ERα*. Inputs came from the total DNA and served as the same ChIP-PCR conditions. (**C**) Bar chart is represented relative histone modification enrichment in the *ERα* promoter. (**D**) Relative binding abilities of SUV39H1 and P300 to the *ERα* promoter. Data were calculated from the corresponding DNA fragments amplified by ChIP-PCR. DNA enrichment was calculated as the ratio of each bound sample divided by the input while the untreated control sample is represented as 1. Columns, mean; Bars, SD; **p < 0.01; ***p < 0.001, significantly different from control.

Histone modification is one of the most important epigenetic mechanisms regulating gene expression and both dietary compounds including EGCG from GTPs and SFN from BSp have shown strong epigenetic regulatory abilities in influencing histone modification processes. We therefore conducted our subsequent experiments to investigate whether this combination regimen can elicit any effect on histone remodeling that may contribute to *ERα* reactivation. We performed ChIP assays to analyze histone modification changes in the *ERα* promoter by detecting several important chromatin markers including transcriptionally active (acetyl-H3, acetyl-H3K9 and acetyl-H4) and inactive (trimethyl-H3K9) markers of chromatin in mouse orthotopic tumors. As shown in Fig. 7A,C, we found that combinatorial treatment with GTPs and BSp can significantly increase enrichment of three histone acetylation chromatin activators, acetyl-H3, acetyl-H3K9 and acetyl-H4, in the *ERα* promoter. This effect was more efficacious in the acetyl-H3 and acetyl-H3K9 chromatin markers combined with TAM than either treatment alone suggesting TAM, acting as an anti-hormone drug, may facilitate histone modification processes in regulation of *ERα* expression, which is consistent with our previous studies indicating TAM is able to interact with epigenetic modulators such as HDACs^7^. Furthermore, dietary GTPs and BSp decreased the binding of trimethyl-H3K9, a transcriptional repressor of histone methylation, in the *ERα* promoter contributing to *ERα* reactivation. These results suggest that our novel combination approach can re-modulate histone modification patterns in the ERα promoter especially when it is combined with TAM treatment, which contributes to *ERα* reactivation in ERα-negative breast cancer cells.

Numerous studies have shown that many epigenetic modulators can interact with other key transcription factors forming a protein complex in the gene promoter that contributes to further chromatin structure changes and gene transcription alteration. For example, the epigenetic modulators, HDAC and DNMT1, can recruit a number of transcriptional repressors to the gene promoters leading to gene silencing^41, 42^. Another important epigenetic factor such as a histone methyltransferase, SUV39H1, is a component of the transcriptional repressor complex that contributes to *ERα* repression^5, 43^. Furthermore, a histone acetyltransferase, P300, acting as a transcriptional co-activator, can switch DNMT1 in this complex and subsequently induce *ERα* transcription^5, 44^. We therefore sought to explore whether our combinatorial treatment of GTPs and BSp can affect the binding of this transcriptional co-repressor/co-activator to the *ERα* promoter, thus influencing *ERα* transcription. We found that this combination treatment can significantly decrease the binding of the transcriptional co-repressor, SUV39H, but increase the binding of the transcriptional co-activator, P300, to the *ERα* promoter (Fig. 7B,D). Collectively, these results suggest that combinatorial treatment of GTPs and BSp can alter the binding abilities of epigenetic transcriptional co-repressor/co-activator to the *ERα* promoter, which leads to histone remodeling and chromatin structure changes around the *ERα* promoter resulting in transcription reactivation of *ERα*.

## Discussion

The use of bioactive dietary components has been widely accepted as an important alternative approach for cancer chemoprevention and therapy. Traditional single-agent dietary approaches, while certainly worthwhile, can have limitations in that these compounds may not be sufficiently efficacious when acting alone or they may require impractical or unsafe levels of consumption to acquire efficacy. A solution to this challenge is combinatorial approaches allowing reduced dosages of natural dietary compounds that, in combination, render greater efficacy. In the current study, we focused on studying combinatorial effects of green tea polyphenol EGCG and broccoli sprouts SFN due to their characteristics of safe use, physiological availability and robust anti-cancer properties compared to other bioactive dietary compounds^5, 6, 18, 24, 32^. Most importantly, EGCG in GTPs and SFN in BSp are considered as potent components of the “epigenetics diet” that can modulate epigenetic pathways leading to reversal of aberrant epigenetic landmarks during carcinogenesis^3, 4, 12^. Previous studies have shown that EGCG and SFN preferably influence epigenetic pathways via direct and/or indirect regulation of DNA methylation and histone modification processes, respectively^16, 17, 23^. The combinatorial administration with EGCG and SFN, acting as a DNA methylation inhibitor and a histone deacetylation inhibitor, respectively, is an attractive approach to overcoming multiple levels of epigenetic abnormality in DNA methylation and chromatin deacetylation. Although similar analyses have been done in a phase I/II clinical trial by using combined epigenetic therapy with azacitidine and entinostat, inhibitors of DNA methylation and histone deacetylation, respectively, in patients with refractory advanced non-small cell lung cancer^45^, epigenetic agents associated with extensive toxicity and low efficacy in solid tumors reduce enthusiasm for wide use these epigenetic drugs in practice^46, 47^. The use of the botanical nutritive has similar favorable biological effects on epigenetic regulation but confers low cytotoxicity, which shows great translational potential in cancer prevention and therapy.

We investigated potential dietary chemopreventive approaches for breast cancer in our current study. Clinically, breast cancers have been classified as either ERα-positive or ERα-negative. ERα status is a useful marker to predict clinical response to endocrine therapy. Patients diagnosed with ERα(−) breast cancer normally have lower survival but higher recurring rates than ERα(+) breast cancer patients due in part to a loss of target therapy such as tamoxifen, which is designed to interrupt the function of ERα. Our previous studies indicate treatment with EGCG or SFN alone beneficially reactivates *ERα* expression in ERα(−) breast cancer cells^5, 24, 32^. We therefore extended our study *in vitro* and *in vivo* to better understand the potential combinatorial effects of green tea polyphenol EGCG and broccoli sprouts SFN in *ERα* reactivation and the potential epigenetic mechanisms underlying these phenomena. More importantly, we sought to explore the preventive or therapeutic properties of this combination regimen and whether it can enhance chemotherapy response to conventional hormone treatment in this refractory breast malignancy.

The present studies have shown a synergistic effect on inhibition of breast cancer growth when EGCG at 20 µM and SFN at 10 µM were combined together at relatively low concentrations without causing toxicity in normal control cells. The concentrations of EGCG and SFN used in this study are physiologically achievable and practical for human study, which are equal to drinking less than half a cup of green tea and consuming ~18 g BSp/serving per day by an adult human, respectively^30, 31^. This result indicates that our combinatorial botanical approach is safe and efficacious in breast cancer prevention and therapy. More importantly, this combinatorial regimen increased cellular response to hormone treatment through activation of *ERα* expression. Although there is a concern that re-expression of *ERα* in ERα(−) breast cancer cells may stimulate ERα-associated signal pathways leading to increased cell proliferation, most of the studies on ERα function show that introduction of *ERα* into ER(−) breast cancer cells can not reconstitute estrogen-dependent tumor growth but rather induce growth inhibition^48^. These conflicting results show that cell-dependent effects of estradiol do not depend solely on *ERα* expression; however, increased *ERα* expression can enhance anti-estrogen chemosensitivity such as to TAM, which is a beneficial change in hormone-resistant breast cancer cells that may apply to future clinical trials.

Further mechanistic studies revealed that combinatorial treatment with EGCG and SFN can induce alterations of gene expression and enzymatic activities of two important epigenetic modulators/enzymes such as DNMT1 and HDAC1 that contribute to *ERα* transcriptional activation. Target studies by knockdown of DNMT1 and HDAC1 expressions (Fig. 4C,D) and evaluation of direct epigenetic effects of additional ECGC/SFN (Fig. S3) on untreated cells further demonstrated that this combinatorial regimen may affect *ERα* transcription efficiency via regulation of epigenetic pathways by directly influencing gene expression of DNMT and HDAC leading to changes in chromatin structures in the *ERα* promoter. In addition to directly influencing epigenetic processes, DNMT1 and HDAC1 can also act as transcriptional factors and recruit a number of transcriptional repressors to the gene promoter region leading to gene silencing^41, 42^. Our *in vitro* ChIP analysis (Fig. 4E,F) indicates that decreased binding abilities of DNMT1 and HDAC1 in the *ERα* promoter in response to EGCG and SFN treatment may impair the recruitment of transcriptional repressor complex to the *ERα* promoter that further render *ERα* reactivation. This has been further proven in our *in vivo* ChIP analysis (Fig. 7) indicating the important roles of histone modification pattern changes on *ERα* reactivation in ERα(−) breast cancer cells. In summary, our results indicate that green tea polyphenol EGCG and broccoli SFN can regulate both genetic and epigenetic mechanisms via, at least in part, directly inhibiting gene expression of two important epigenetic modulators, DNMT1 and HDAC1, leading to an opened chromatin structure and altered transcriptional factor binding status in the *ERα* promoter that may contribute to *ERα* transcriptional activation in ERα(−) breast cancer cells.

We also tested this dietary regimen in an ER(−) orthotopic xenograft mouse model system and found convincing results in inhibition of tumor growth of orthotopic xenografts in mouse mammary glands when orally-fed GTPs and BSp diets were administered. This inhibitory effect was further strengthened when a conventional anti-hormone treatment, TAM, was introduced, suggesting this dietary combinatorial approach can resensitize TAM-associated anticancer capacity through at least in part, epigenetic reactivation of *ERα* expression in mouse orthotopic tumors. Our further observations revealed that gene expression as well as enzymatic activities of DNMT1 and HDAC1 may also play an important role in *ERα* transcription regulation *in vivo*. Altered histone modification patterns and binding abilities of co-activator/co-repressor in the *ERα* promoter may also contribute to *ERα* reactivation. The dietary concentrations used in mouse studies were 0.3% GTP in drinking water and/or 13% BSp in formulated diet, which are equivalent to 1–2 cups of green tea (1.5 mg EGCG/ml water) and 133 g (~2 cups) broccoli sprout/per day (8.45 uM SFN/g diet) for human consumption^39, 40^. Therefore, the *in vivo* concentrations of GTPs and BSp also represent practical consumption levels in the human diet. Collectively, these studies indicate that combined GTPs and BSp are highly effective in inhibiting ER(−) breast cancer development by re-sensitizing TAM-induced anti-cancer capacity through epigenetic activation of *ERα* expression.

Our findings provide important observations for the use of a combinatorial approach of dietary green tea polyphenol EGCG and broccoli sprouts SFN in resensitization of anti-hormone chemotherapy of TAM by inducing epigenetic *ERα* reactivation in ERα-negative breast cancer *in vitro* and *in vivo*. This novel dietary combinatorial regimen has high translational potential by providing safe and physiologically achievable doses, which could be potentially useful in future clinical human studies. The combinatorial approach is novel because it is necessary to induce maximal effects on neutralizing aberrant epigenetic profiles against breast cancers. The involvement of epigenetic control of this dietary combinatorial treatment in regulating *ERα* expression and subsequent enhanced efficacy of TAM chemotherapy provides new avenues for prevention and therapeutic application for *de novo* hormone-resistant breast cancer patients. Future efforts will aim at determining the effectiveness of these combined botanicals on human breast cancers.

## Materials and Methods

### Cell culture and treatment

Breast cancer cell lines including ERα-positive MCF-7 and ERα-negative MDA-MB-231 and MDA-MB-157 cells were obtained from American Type Culture Collection (ATCC). Breast cancer cells were grown in phenol-red–free medium DMEM (Invitrogen, Carlsbad, CA) supplemented with 10% dextran-charcoal–stripped fetal bovine serum (Atlanta Biologicals, Lawrenceville, GA) and 1% penicillin/streptomycin (Mediatech, Herndon, VA). Cells were maintained in a humidified environment of 5% CO_2_ and 95% air at 37 °C and treated with the indicated concentrations of EGCG and/or SFN for a total of 3 days to evaluate the combinatorial effect of EGCG and SFN (Sigma, St. Louis, MO) treatment. To observe the effects of 17β-estradiol (E_2_) (Sigma) and tamoxifen (TAM) (Sigma) on *ERα* expression, EGCG- and SFN-pretreated MDA-MB-231 cells were then exposed with or without 10 nM of E_2_ or 1 µM TAM for an extra two days, respectively. Control cells received equal amounts of DMSO (Sigma) in the medium. The culture medium was replaced every 24 h for the duration of the experiment.

### MTT assay for cell viability

Aliquots of cells were seeded in triplicate in 96-well plates and treated with the indicated concentrations of EGCG and/or SFN for 3 days to determine the effects of combinatorial treatment on cell viability. The MTT reagent (Sigma) was added to the culture medium to achieve a final concentration of 1 mg/ml followed by 4 h incubation at 37 °C until purple precipitates were visible. The media were aspirated and the cells were dissolved in 100 µl DMSO. The absorbance of the cell lysates was measured at 570 nm by an Epoch Microplate Spectrophotometer (BioTek, Winooski, VT) as done previously^5–8^.

### Quantitative real-time PCR

ERα-negative breast cancer MDA-MB-231 and MDA-MB-157 cells were cultured and treated as described above. Total RNA from cells was extracted using the RNeasy kit (Qiagen, Valencia, CA) according to the manufacturer’s instructions and reversely transcribed to cDNA using iScript cDNA Synthesis kit (Bio-Rad, Hercules, CA Biorad) as performed previously^5–8^. Specific gene primers for *ERα*, *DNMT1*, *HDAC1* and *glyceraldehyde-3-phosphate dehydrogenase* (*GAPDH*) were provided by Integrated DNA Technologies (Coralville, Iowa) as following: 5′-CAAGCCCGCTCATGATCAA-3′ (F), 5′-CTGATCATGGAGGGTCAAATCCAC-3′ (R) for *ERα*; 5′-ACCGCTTCTACTTCCTCGAGGCCTA-3′ (F), 5′-GTTGCAGTCCTCTGTGAACACTGTGG-3′ (R) for *DNMT1*; 5′-ATGGACGATCTGTTTCCCCT-3′ (F), 5′-CGGTTTACTCGGCAGATCTT-3′ (R) for *HDAC1* and 5′-ACCACAGTCCATGCCATCAC-3′ (F), 5′-TCCACCACCCTGTTGCTGTA-3′ (R) for *GAPDH*. In the real-time PCR step, gene expression were performed in triplicate and analyzed by real-time PCR using SYBR GreenER qPCR Supermix (Invitrogen) in a BioRad CFX Connect Real-time System. Thermal cycling was initiated at 94 °C for 4 min followed by 35 cycles of PCR (94 °C, 15 s; 60 °C, 30 s). GAPDH was used as an endogenous control, and vehicle control was used as a calibrator. The relative changes of gene expression were calculated using the following formula: fold change in gene expression, 2^−ΔΔCt^ = 2^−{ΔCt (treated samples) − ΔCt (untreated control samples)}^, where ΔCt = Ct (test gene) − Ct (GAPDH) and Ct represents threshold cycle number.

### Western blot analysis

Tissue cultured protein extracts were prepared by using RIPA Lysis Buffer (Upstate Biotechnology, Charlottesville, VA) and proteins from mouse xenograft tumors were homogenized and extracted with T-PER Tissue Protein Extraction Reagent (Thermo Scientific, Waltham, MA) according to the manufacturers’ protocols. Proteins were electrophoresed in Biorad SDS-polyacrylamide ready gels and transferred onto nitrocellulose membranes. Membranes were then probed with different antibodies to ERα (Ab-12; NeoMarkers, Fremont, CA), HDAC1 (H11; Santa Cruz Biotechnology) and DNMT1 (ab13537; Abcam, San Francisco, CA) respectively, and each membrane was stripped and reprobed with Beta-actin antibody (13E5, Cell Signaling Technology, Boston, MA) as loading control. Immunoreactive bands were visualized using the enhanced chemiluminescence detection system (Santa Cruz Biotechnology) and documented by a ChemiDoc™ Imaging Systems (Biorad).

### HDACs and DNMTs activity assay

Cultured cells were harvested at the indicated time points and nuclear proteins from breast cancer MDA-MB-231 and MDA-MB-157 cells were extracted by NE-PER Nuclear and Cytoplasmic Extraction Reagents (Thermo Scientific). The activities of HDACs and DNMTs were evaluated by EpiQuik HDAC Activity/Inhibition Assay Kit and EpiQuik DNMT Activity/Inhibition Assay Kit (Epigentek, Brooklyn, NY) according to the manufacturer’s protocols, respectively, as done previously^24^. The enzymatic activities of HDACs and DNMTs were colorimetrically demonstrated and detected by an Epoch Microplate Spectrophotometer at 450 nm.

### RNA interference

Validated siRNA for *DNMT1* (ID: S4215) and *HDAC1* (ID: S73) and the positive/negative control RNAi (Thermo Scientific, Applied Biosystems) were transfected into ERα-negative breast cancer MDA-MB-231 cells by the indicated treatment using the Silencer siRNA Transfection II Kit (Thermo Scientific) according to the protocols provided by the manufacturer^24^. Relevant gene expression and enzymatic activities were detected ~72 h after transfection.

### Animal models, dietary and experimental designs

A breast cancer orthotopic mouse model was used in this study. Virgin female immunodeficiency Nu/Nu Nude mice (Crl:NU-*Foxn1nu*) at 4-6 weeks of age were obtained from Charles River Laboratories (Wilmington, MA) and housed in the Animal Resource Facility of the University of Alabama at Birmingham (UAB) under the following conditions: 12-h dark/12-h light cycle, 24 ± 2 °C temperatures, and 50 ± 10% humidity.

An online power calculator (http://powerandsamplesize.com/) was used to calculate the power/sample size by 2-proportion comparison based on our *in vitro* data. A sample size of 5 mice/group will give us 95% power for detecting beneficial botanical effects for a one-sided test at significance level of 0.05 (α = 0.05).

After one week of acclimatization, Nu/Nu Nude mice were randomly divided into six experimental groups (5 mice each): 1) Control group: mice were fed with AIN-93G diet; 2) Green tea polyphenols (GTPs) diet: 0.3% (w/v, 3 mg/ml) of GTPs (Sunphenon 90D, Taiyo International, Inc., Minneapolis, Minnesota) were administered in drinking water; 3) Broccoli sprout (BSp) diet: mice were fed with 13% of BSp diet (w/w, modified AIN-93G diet supplemented with 13% broccoli sprouts; TestDiet, St. Louis, MO); 4) Combination diet: mice were fed with both 0.3% of GTPs and 13% of BSp diets. 5) Combination diet with tamoxifen (TAM): mice were fed with combination diet plus TAM treatment for 3 wks after two wks of post-injection (25 mg/pellet with 21 days release, subcutaneous implantation under the neck area, Innovative Research of America, Aarasota, FL) 6) TAM group: mice were administered control diet and received TAM treatment as described above.

Relevant diet treatments were provided from two weeks prior to injection and the mice continued to receive the corresponding experimental diets throughout the study. To determine the *in vivo* efficacy of combinatorial treatment with GTPs and BSp diets on ERα reactivation and subsequent chemosensitization to estrogen antagonist, TAM, in human ERα-negative breast tumor xenografts, exponentially growing MDA-MB-231 cells were mixed at a 1:1 ratio with Matrigel (Becton Dickinson). A 100 µl aliquot of exponentially growing breast cancer MDA-MB-231 cells (2 × 10^6^) was injected orthotopically into the second or third pair of the right thoracic mammary fat pad of each mouse.

Tumor sizes and body weight were measured weekly. Tumor volumes were calculated using the formula: tumor volume (cm^3^) = (length × width^2^) × 0.523^7, 24^. The experiment was terminated when the mean of tumor diameter in the control mice exceeded 1.0 cm. At the end of the experiment, the primary breast tumors were excised, weighed and appropriately stored in liquid nitrogen for further analysis. A tumor slice from each primary tumor tissue was carefully dissected and fixed in 10% buffer-neutralized formalin for histology and immunohistochemistry. Tumor specimens were snap frozen in liquid nitrogen for further studies such as RNA and protein extraction. Animal procedures were reviewed and approved by UAB IACUC (Animal Project Number: 110109327). All experiments and procedures were performed in accordance with the guidelines of the Institutional Animal Care and Use Committee (IACUC) at UAB.

### Immunohistochemical determination of tumor cell proliferation

Primary breast tumor tissues were dissected and fixed in 10% buffer-neutralized formalin for histology and immunohistochemistry. Tumor slices (5 µm thick) were deparaffinized and rehydrated in a series of graded alcohols. Antigens were retrieved in boiling 10 mmol/L sodium citrate buffer (pH 6.0) for 20 min. The slides were then washed in PBS and blocked with 1% bovine serum albumin. After blocking, the slides were incubated with anti-proliferating cell nuclear antigen (PCNA) (Cell Signaling Technology) for 2 h at room temperature. After washing in PBS, the sections were incubated with biotinylated secondary antibody for 45 min followed by horseradish peroxidase-conjugated streptavidin, incubated with diaminobenzidine substrate and counterstained with hematoxylin. Representative photographs were taken and the numbers of PCNA-positive cells were detected and counted by using an Olympus BX41 microscope fitted with a Q-color 5 Olympus camera.

### Chromatin Immunoprecipitation (ChIP) Assay

Breast cancer MDA-MB-231 or MDA-MB-157 cells were treated with EGCG and SFN alone or in combination as indicated previously. Approximately 2 × 10^6^ cells were cross-linked with a 1% final concentration of formaldehyde (37%, Fisher Chemicals, Fairlawn, NJ) for 10 min at 37 °C. ChIP assays were performed with the EZ Chromatin Immunoprecipitation (EZ ChIP^TM^) assay kit according to the manufacturer’s protocol (Upstate Biotechnology) as described previously^5–7^. For tissue-based ChIP assay, snap-frozen mouse orthotopic tumor (~20 mg) was thawed and used for each group in ChIP assay. Breast tumor tissues were cross-linked with a 1.5% final concentration of formaldehyde, degradated and sonicated followed by the protocol of chromatin preparation from tissues for chromatin immunoprecipitation (ChIP) (Abcam). Subsequent ChIP assays were performed with the EZ Chromatin Immunoprecipitation (EZ ChIP^TM^) assay kit according to the manufacturer’s protocol as described previously^5–7^. The epigenetic antibodies used in the ChIP assays were ChIP-validated acetyl-histone H3 (H3ac, Upstate Biotechnology), acetyl-histone H3-Lys9 (H3K9ac, Upstate Biotechnology), acetyl-histone H4 (H4ac, Upstate Biotechnology), trimethyl-histone H3-Lys9 (H3K9me3, Upstate Biotechnology), SUV39H1 (Santa Cruz Biotechnology), P300 (Santa Cruz Biotechnology), HDAC1 (Abcam) and DNMT1 (Abcam). ChIP-purified DNA was amplified by standard PCR using primers specific for the *ERα* promoter yielding a 150 bp fragment: sense, 5′-GAACCGTCCGCAGCTCAAGATC-3′ and anti-sense, 5′- GTCTGACCGTAGACCTGCGCGTTG -3′. PCR amplification was performed using the 2 × PCR Master Mix (Promega, Madison, WI) and the reaction was initiated at 94 °C for 4 min followed by 30 cycles of PCR (94 °C, 30 s; 56 °C, 30 s; 72 °C, 1 min), and extended at 72 °C for 5 min. After amplification, PCR products were separated on 1.5% agarose gels and visualized by ethidium bromide fluorescence using Kodak 1D 3.6.1 image software (Eastman Kodak Company, Rochester, NY). Quantitative data were analyzed using the Sequence Detection System software version 2.1 (PE Applied Biosystems, Foster City, CA).

### Statistical analyses

Statistical significance between treatment and control groups was evaluated by one-way ANOVA followed by Tukey’s test for multiple comparisons by using GraphPad Prism 5.00 version. Values were presented as mean ± SD (standard deviation) and *P* < 0.05 was considered statistically significant. Western-blot protein density was analyzed by Image J software developed by NIH. The synergistic effects between the treatments of EGCG and SFN were determined by an online software, CompuSyn, in which Combination Index (CI) <1, = 1 and >1 indicate synergism, additive effect and antagonism, respectively^49^.

### Ethics approval and consent to participate

Animal procedures in this study were approved by UAB IACUC (Animal Project Number: 110109327).

## Electronic supplementary material

Supplementary Figures
