# Supplementary material for: Combinatorial bioactive botanicals re-sensitize tamoxifen treatment in ER-negative breast cancer via epigenetic reactivation of ERα expression
Source: Sci Rep. 2017 Aug 24;7:9345. doi: 10.1038/s41598-017-09764-3 (PMC5570897; doi:10.1038/s41598-017-09764-3)
Supplement: Supplementary file 1 — Supplementary Figures [file 41598_2017_9764_MOESM1_ESM.pdf]

Supplementary Figures

Combinatorial bioactive botanicals re-sensitize tamoxifen treatment in ER-negative breast cancer via epigenetic reactivation of ERα expression

Yuanyuan Li[1,2,3,*], Syed M. Meeran[4,] and Trygve O. Tollefsbol[1,2,3,5]

[1]Department of Biology, University of Alabama at Birmingham, Birmingham, Alabama, 35294, USA

[2]Comprehensive Cancer Center, University of Alabama at Birmingham, Birmingham, Alabama, 35294, USA

[3]Nutrition Obesity Research Center, University of Alabama at Birmingham, Birmingham, Alabama, 35294, USA

[4]Department of Biochemistry, CSIR-Central Food technological Research Institute, Mysore, 570019, India.

[5]Comprehensive Center for Healthy Aging, University of Alabama at Birmingham, Birmingham, Alabama, 35294, USA

[*]Address correspondence to Yuanyuan Li, 1300 University Blvd, CH175, Birmingham, AL, 35294-1170; Tel: +1-205-934-4587; Fax: +1-205-975-6097; E-mail: lyy@uab.edu

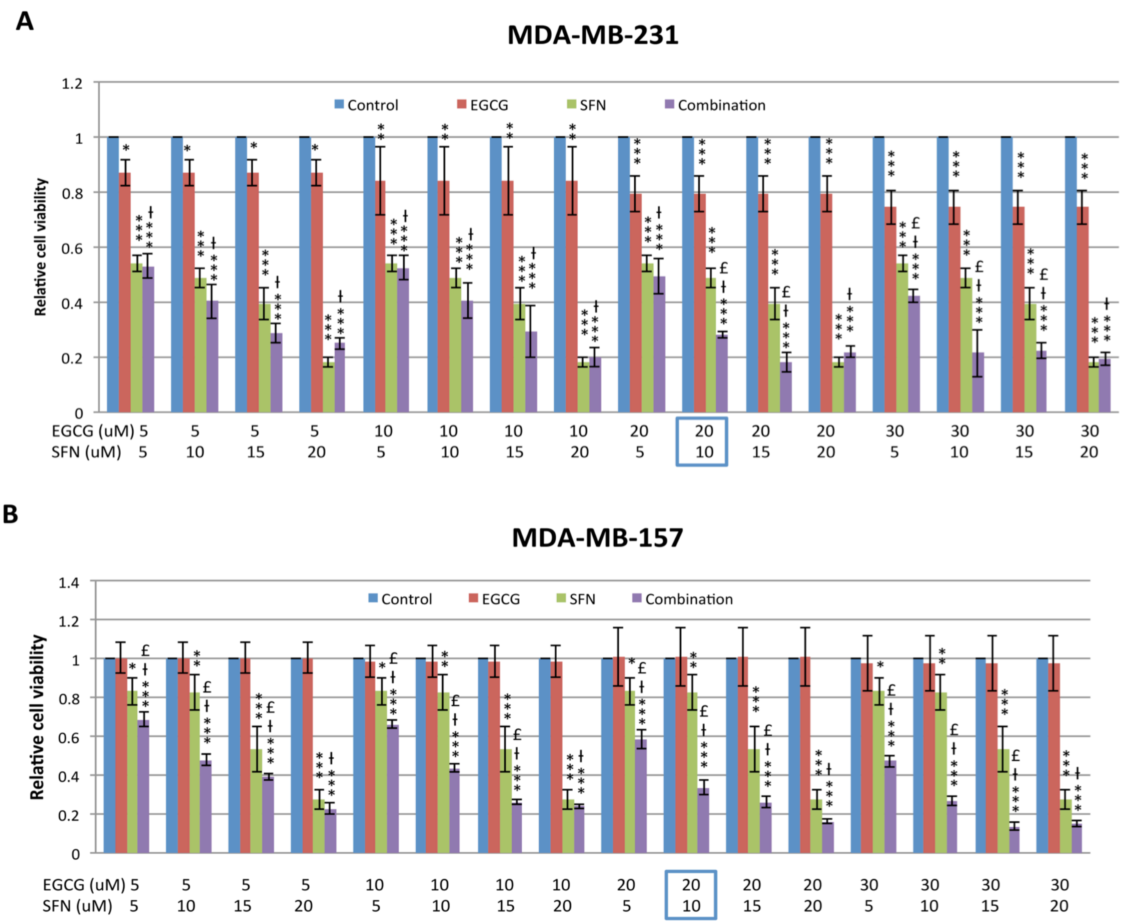

Fig. S1 Determination of an optimal concentration of EGCG and SFN combinatorial treatment by growth inhibitory effects. MDA-MB-231 (A) and MDA-MB-157 cells (B) cells were treated with different concentrations of EGCG and SFN, alone or in combination for 3 days. The treatment concentrations of EGCG or SFN alone or in combination are illustrated at the bottom of each graph. EGCG at 20 µM EGCG and 10 µM SFN represented the optimal concentrations (blue square) of these two compounds with physiological availability as well as potential synergistic effects on breast cancer growth inhibition. Columns, mean; Bars, SE; *, p<0.05; **, p<0.01; ***, p < 0.001,

significantly different from control; £, $p < 0.001$, significantly different from EGCG; †, $p < 0.05$, significantly different from SFN.

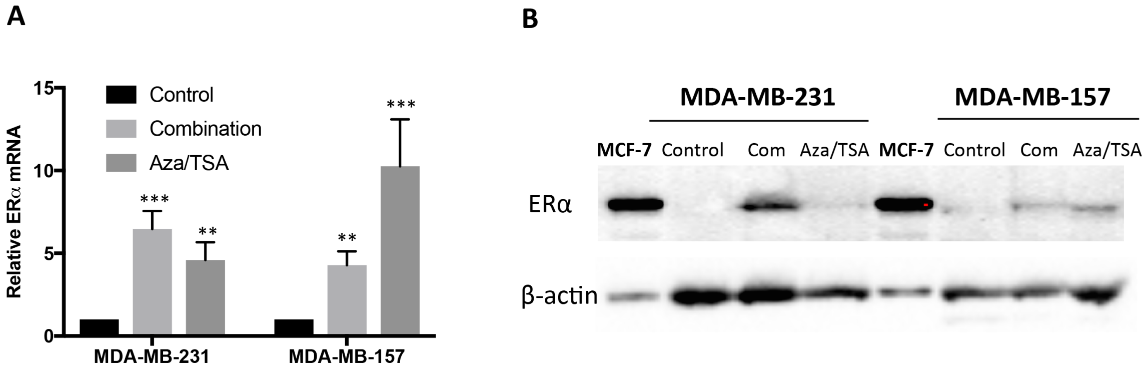

Fig. S2 Combined treatment with EGCG and SFN or 5-azacytidine (5-aza) and Trichostatin A (TSA) induced ERα reactivation in ERα-negative breast cancer cells. A, ERα-negative breast cancer MDA-MB-231 and MDA-MB-157 cells were treated with either EGCG and SFN, or 5-aza (2 μM) and TSA (100ng/ml) as done previously and relative *ERα* mRNA expression was analyzed by quantitative real-time PCR. B, Protein expression of ERα in MDA-MB-231 and MDA-MB-157 cells. MCF-7 cells served as a positive control. The full-length blots were shown in the Fig. S4. Data were in triplicate from three independent experiments and normalized to internal control and calibrated to levels in untreated samples. Com, EGCG and SFN in combination; Aza/TSA, 5-azacytidin and Trichostatin A in combination; Columns, mean; Bars, SD; *, $p < 0.05$; **, $p < 0.01$; ***, $p < 0.001$, significantly different from control.

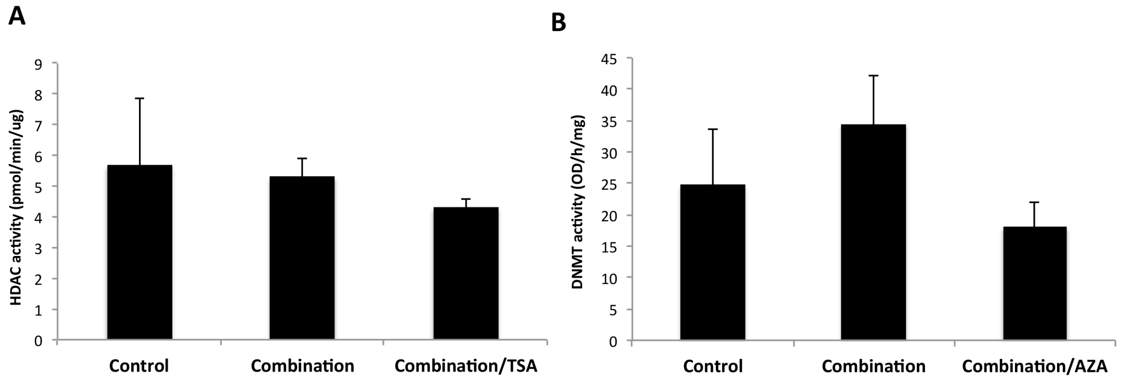

Fig. S3 Enzymatic activities of HDAC1 and DNMT1 by post-EGCG and SFN treatment. A. HDAC enzymatic activity. B. DNMTs enzymatic activity. EGCG and SFN were added to untreated nuclear extract from MDA-MB-231 cells to evaluate direct inhibitory effects. The HDAC and DNMT activity assays were performed according to the manufacturer's protocols. Additional 5-aza and TSA were used as the controls. The values of enzymatic activities of HDACs and DNMTs are the means of three independent experiments.

Figure 2C

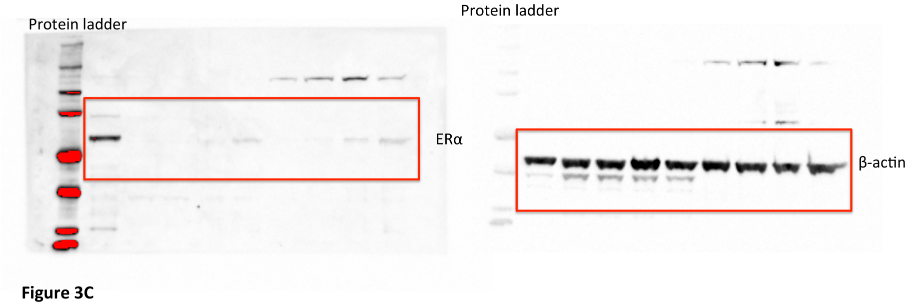

Figure 3C

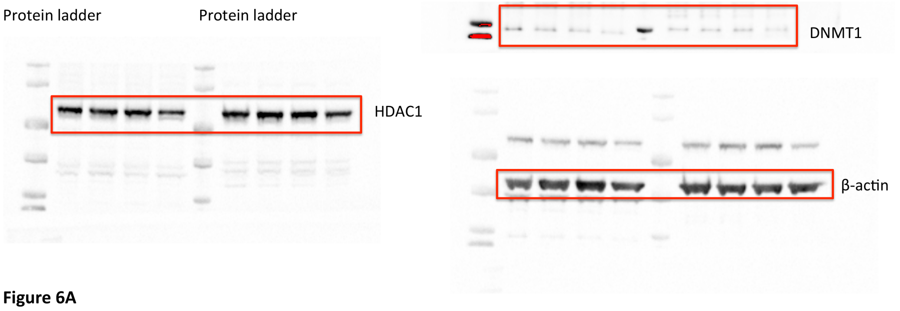

Figure 6A

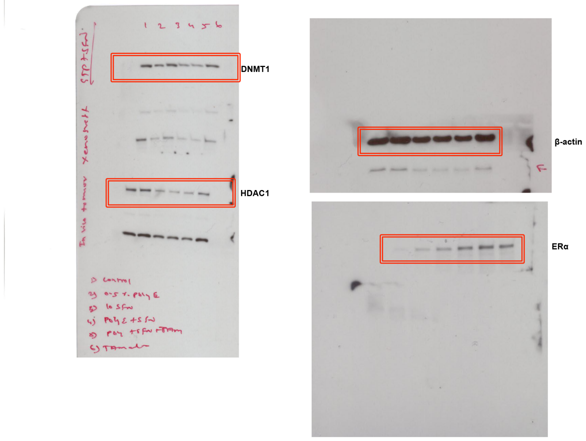

Figure 7A and 7B

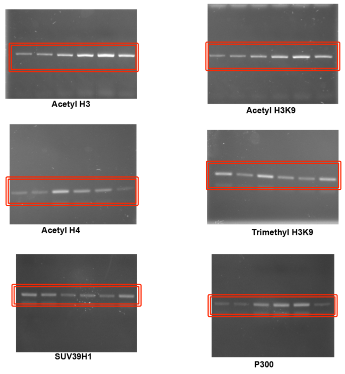

Figure S2B

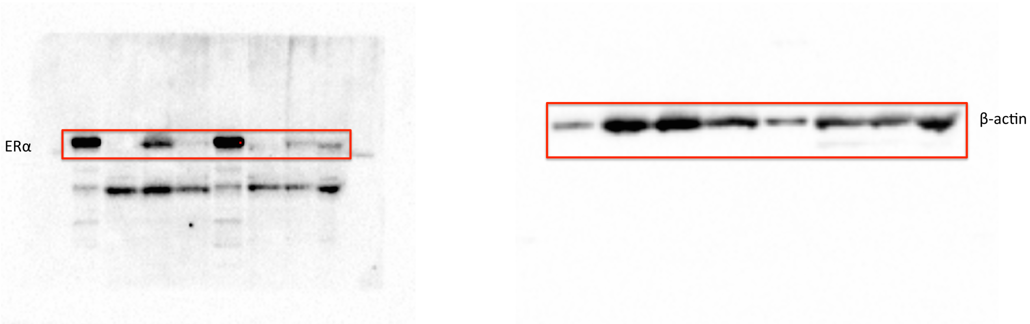

Fig. S4 Uncropped figures
